# Post-Traumatic Growth of Nurses Who Faced the COVID-19 Epidemic and Its Correlation With Professional Self-Identity and Social Support

**DOI:** 10.3389/fpsyt.2021.562938

**Published:** 2022-01-14

**Authors:** Yuanyuan Mo, Pinyue Tao, Guiying Liu, Lin Chen, Gaopeng Li, Shuyu Lu, Guining Zhang, Rong Liang, Huiqiao Huang

**Affiliations:** ^1^Department of Psychiatry, The Second Affiliated Hospital of Guangxi Medical University, Nanning, China; ^2^Department of Nursing, The Second Affiliated Hospital of Guangxi Medical University, Nanning, China; ^3^Department of Nursing, Nursing College of Guangxi Medical University, Nanning, China

**Keywords:** COVID-19, nurses, professional self-identity, social support, Hubei, cross-sectional survey, post-traumatic growth

## Abstract

**Objective:**

To investigate post-traumatic growth (PTG) and analyze its correlation with professional self-identity and social support in Chinese nurses who faced the coronavirus disease 2019 (COVID-19) epidemic.

**Methods:**

A cross-sectional descriptive design was used in this study. An online questionnaire was completed by 266 nurses who faced the COVID-19 emergency in Hubei Province, China. The Post-traumatic Growth Inventory (PTGI), Professional Self-identity Scale, and Perceived Social Support Scale were used to assess the level of PTG, professional self-identity, and social support. Descriptive, univariate analysis and multiple regression analyses were used in exploring related influencing factors.

**Results:**

Participants' mean scores were 96.26 (SD = 21.57) for PTG, 115.30 (SD = 20.82) for professional self-identification, and 66.27 (SD = 12.90) for social support. Multiple regression analysis showed that nurses from other provinces moving to support Hubei Province, professional self-identity, and social support were the main factors affecting nurse stress (*p* = 0.014, < 0.001, and 0.017, respectively). Professional self-identity and social support were positively correlated with PTG (*r* = 0.720 and 0.620, respectively).

**Conclusions:**

There was a phenomenon of PTG when the nurses faced COVID-19 in Hubei Province. Providing an active coping style helps to improve the level of PTG.

## Introduction

The current pandemic is caused by a novel coronavirus that has been dubbed COVID-19 (1). On January 30, 2020, the World Health Organization (WHO) declared the COVID-19 outbreak a global health emergency (2). As the epidemic spread around the world, on March 11, 2020, the WHO officially classified COVID-19 as a pandemic (3).

“Nurses are the backbone of any health system. Today, many nurses find themselves on the frontline in the battle against COVID-19,” said Dr. Tedros Adhanom Ghebreyesus (4). A total of 28,600 nurses had sent to Hubei Province to fight against the COVID-19 epidemic in China (5). Nurses on the frontline in this event are showing commitment and compassion, but they are putting their lives at risk in the course of their duties (6). The COVID-19 outbreak has been even more devastating. More than 3,000 medical workers in China were infected (7), over 8,000 in Italy (8), and over 19,000 in Spain (9). The unfolding emergency caused by COVID-19 is putting nursing services under intense pressure.

A systematic review concluded that high prevalence of post-traumatic stress symptoms was related to the COVID-19 pandemic among healthcare workers and summarized potential predictors, such as young age, female, and lack of social support (10). Distressing or harmful events can result in negative outcomes, such as post-traumatic stress disorder (PTSD) symptoms (11), but they can also lead to positive post-trauma changes, experiences termed post-traumatic growth (PTG). PTG, first proposed by Tedeschi and Calhoun in 1996 (12), is defined as a significant positive change in an individual's life as a consequence of exposure to a challenging or traumatic event. According to Tedeschi and Calhoun's theory, social support, as a positive external resource, could facilitate PTG. However, whether the positive relationship between social support and PTG in persons who have experienced trauma directly should be investigated further.

Nurses have always played an important role in public health (13). Maintaining the mental health of nursing staff is essential to controlling infectious diseases (14, 15). Certain investigations have focused on post-traumatic growth in nurses in China, but mainly on the analysis of influencing factors. This study aimed to explore the level of PTG among frontline nurses and analyzed its correlations with professional self-identity and social support. On the basis of Tedeschi and Calhoun's model of PTG, we hypothesized that the phenomenon of PTG among frontline nurses existed and was correlated with professional self-identity and social support.

## Materials and Methods

### Study Design

The study design used a descriptive cross-sectional survey. Data were collected from February 1, 2020 to April 30, 2020.

### Participants and Sampling

Nurses who faced the COVID-19 epidemic in Hubei Province were selected for the survey. The inclusion criteria were as follows: licensed nurses, nurses who were working against COVID-19, those without other traumatic events (e.g., sudden deaths of immediate family members and traffic accidents) in the past 6 months, and those who volunteered to participate in this study. A total of 270 frontline nurses completed the questionnaire, and 266 respondents provided usable data.

The study involving human participants were reviewed and approved by the Ethics Committee of the Second affiliated hospital of Guangxi Medical University (No. 2020-KY0005). All subjects had signed an informed consent before the study was initiated. To protect the respondents' privacy, the survey was conducted anonymously.

### Measurement Tools

#### Basic Information Survey Form

A questionnaire was developed to collect basic information, including gender, age, nursing experience, marital status, children, education, staff title, specialty, whether working locally, current working place, patient's disease severity, whether volunteered to participate in support work, whether regretted participating in the support work, support duration, and working hours per day.

#### Assessment of Post-Traumatic Growth

The Post-traumatic Growth Inventory (PTGI) developed by Tedeschi and Calhoun is currently widely used to evaluate post-traumatic growth (12). The scale consists of five major domains: relating to others, new possibilities, personal strength, spiritual change, and appreciation of life. A total of 21 entries use a six-point scoring method, with the total score in the range of 0–105 points. In this study, the Cronbach's alpha coefficient for the total PTGI was 0.972. The subscales' alpha coefficients were as follows: Relating to Others = 0.789, New Possibilities = 0.856, Personal Strength = 0.944, Spiritual Change = 0.897, and Appreciation of Life = 0.943.

#### Assessment of Professional Self-Identity

A nurse professional self-identity scale was developed by professor Liu (16). The scale consists of five dimensions: professional cognitive, professional social support, professional social skills, coping with setbacks, and professional self-reflection, with 30 entries using a five-point scoring method, with the total score in the range of 30–150 points. The higher the score, the greater the professional self-identity. The Cronbach's α value is 0.938.

#### Assessment of Social Support

Zimet et al. developed the Perceived Social Support Scale (17), and Jiang introduced it to China and conducted cultural commissioning (18). The Chinese version of the Perceived Social Support Scale has good reliability and validity. The scale consists of two dimensions: in-family support (four entries) and out-of-family support (eight entries), with 12 entries using a seven-point scoring method, with the total score in the range of 12–84 points. The scores of social support were analyzed as follows: low level 12–28, moderate level 29–57, and high level 58–84. The higher the score, the greater the pressure load. The Cronbach's α value was 0.936.

#### Data Collection

Online survey (via a questionnaire website platform) was sent to the nurse managers in Hubei Province who worked against COVID-19. The managers were asked to forward the survey to nurses. Participants could complete the questionnaire via a computer or mobile phone, which can open a web link or scan a Quick Response code.

#### Data Analysis

Data analysis was performed with IBM SPSS Statistics for Windows (version 21.0, IBM Corp., Armonk, NY, USA), with two-tailed *p* < 0.05 considered statistically significant. Descriptive statistical methods were used to describe the basic characteristics and to assess the level of PTG, professional self-identity, and social support among the participants. Counting data were expressed as frequency and percentage, and the measurement data were expressed as $\bar{x}$ ± s. Two independent samples *t*-test was used for comparison between two groups and one-way ANOVA was used for comparison between multiple groups. Taking the score of PTGI as the dependent variable and all the other indicators as the independent variables, univariate analysis and multiple regression analysis were performed to identify the main influencing factors of clinical frontline nurses' PTG. Data with a value of *p* < 0.05 were considered statistically significant. Pearson correlation analysis was used to explore the correlation between nurses' PTG, professional self-identity, and social support.

## Results

The participants consisted of 266 nurses, the average age of the respondents was 32.34 (SD = 6.01) years, the average length of nursing experience was 11.35 (SD = 3.60) years, the average supporting day was 48.36 (SD = 12.70) days, and the average working hours per day was 5.91 (SD = 1.25) h. More basic information is shown in Table 1.

**Table 1:** Basic information survey form (*N* = 266).

**Variables**	***n* (100%)**
**Gender**	
Male	24 (9.02%)
Female	242 (90.98%)
**Marital statuses**	
Married	145 (54.51%)
Unmarried	119 (44.74%)
Divorced or others	2 (0.75%)
**Children**	
Yes	136 (51.13%)
No	130 (48.87%)
**Education**	
Junior college or below	55 (20.67%)
Undergraduate	205 (77.07%)
Master	6 (2.26%)
**Staff title**	
Junior	147 (55.26%)
Middle	93 (34.96%)
Sub-senior	26 (9.78%)
Senior	0 (0.00%)
**Specialty**
Department of Respiration	39 (14.66%)
Department of Infectious disease	27 (10.15%)
Department of Emergency	54 (20.30%)
Intensive Care Unit	60 (22.56%)
Others	54 (20.30%)
**Whether nurses were from other provinces to support Hubei Province**	
Yes	214 (80.45%)
No	52 (19.55%)
**Current working place**	
Temporary treatment centers	115 (43.23%)
Designated hospitals	151 (56.77%)
**Patient's disease status of care**	
Suspected	53 (19.92%)
Mild	38 (14.29%)
Common	73 (27.44%)
Severe	65 (24.44%)
Critically ill	37 (13.91%)
**Volunteered to participate in the support work**	
Yes	266 (100.00%)
No	0 (0.00%)
**Regret to participate in the support work**	
Yes	0 (0.00%)
No	266 (100.00%)

The participants' mean score for PTG was 96.26 (SD = 21.57). The subscales' mean score of PTG was as follows: Relating to Others = 31.75 (SD = 7.41), New Possibilities = 22.01 (SD = 5.58), Personal Strength = 18.87 (SD = 4.48), Spiritual Change = 9.48 (SD = 2.22), and Appreciation of Life = 14.08 (SD = 3.29). The participants' mean score for professional self-identity was 115.30 (SD = 20.82). The participants′ mean score for social support was 66.27 (SD = 12.90). The scores of each dimension are shown in Table 2.

**Table 2:** Scores of PTG, Professional Self-identity, and Social Support (*N* = 266).

**Items**	**Mean (SD)**
**PTG**	96.26 (21.57)
Relating to others	31.75 (7.41)
New possibilities	22.01 (5.58)
Personal strength	18.87 (4.48)
Spiritual change	9.48 (2.22)
Appreciation of life	14.08 (3.29)
**Professional self-identity**	115.30 (20.82)
Professional cognitive	34.08 (6.99)
Professional social support	23.82 (4.07)
Professional social skills	21.99 (4.71)
Coping with setbacks	23.66 (3.97)
Professional self-reflection	11.76 (2.15)
**Social support**	66.27 (12.90)
In-family support	22.24 (4.89)
Out-of-family support	44.04 (8.61)

Univariate analysis of the PTG of frontline nurses showed that different education profiles, marital statuses, fertility statuses, whether nurses were from other provinces to support Hubei Province, and working hours per day affected nurses' PTG (*p* < 0.05), as follows (Table 3).

**Table 3:** Univariate analysis of the PTG of nurses (x ± s, *N* = 266).

**Items**	**Classification**	***n* (%)**	**PTG (Mean ±SD)**	**Statistics**	***p***
Education	Junior college or below	55 (20.68)	94.41 ± 20.25	*F* = 2.727	0.045
	Undergraduate	205 (77.07)	96.77 ± 21.75		
	Master	6 (2.26)	104.83 ± 16.27		
Marriage	Unmarried	119 (44.74)	92.38 ± 22.82	*F* = 5.695	0.004
	Married	145 (54.51)	99.82 ± 19.84		
	Divorce or others	2 (0.75)	69.00 ± 3.53		
Children	Yes	130 (48.87)	92.51 ± 22.97	*t* = 7.893	0.005
	No	136 (51.13)	99.85 ± 19.55		
Whether nurses were from other provinces to support Hubei Province	Yes	214 (80.45)	93.89 ± 22.64	*t* = 13.820	0.001
	No	52 (19.55)	106.00 ± 12.53		
Working hours per day	<6 h	130 (48.87)	92.51 ± 22.97	*F* = 4.220	0.016
	6–8 h	116 (43.61)	100.41 ± 18.08		
	>8 h	20 (7.52)	96.55 ± 26.94		

*Only statistically significant results are listed*.

To determine the best predictors of PTG, multiple regression analysis was conducted. When the PTG was used as a dependent variable, the single factor analysis of the PTG was statistically significant in education (junior college or below = 1, undergraduate = 2, master = 3), marital status (married = 1, unmarried = 2, divorced or widowed = 3), children (yes = 1, no = 2), whether nurses moved from other provinces to support Hubei Province (yes = 1, no = 2), working hours per day (6 h = 1, >6 h and 8 h = 2, >8 h = 3). The total scores of professional self-identity and social support (substituting the actual value) were independent variables for multiple linear regression analysis. The results showed that whether nurses moved from other provinces to support Hubei Province, professional self-identity, and social support were the main factors influencing the PTG of nurses assisting in the fight against COVID-19, possibly explaining 53.3% of the total variation, as follows (Table 4).

**Table 4:** Multiple-factor analysis of PTG on nurses (x ± s, *N* = 266).

**Dependent variable**	**Regression coefficient**	**SE**	**Standardized regression coefficient**	***t*-value**	***p*-Value**
Constant	7.727	8.376		0.922	0.357
Whether nurses were from other provinces to support Hubei Province	5.781	2.334	0.107	2.477	0.014
Professional self-identity	0.589	0.077	0.569	7.635	0.000
Social support	0.299	0.124	0.179	2.412	0.017

*R^2^ = 0.546, adjusted R^2^ = 0.533, F = 44.255, p < 0.001*.

According to Pearson's correlation analysis, the professional self-identity, and social support were positively correlated with PTG (the *r* values were 0.720, 0.620). The correlations of each dimension were as follows (Table 5).

**Table 5:** Correlation analysis of PTG with professional self-identity and social support (*N* = 266).

	**Professional self-identity**	**Social support**
PTG	0.720^a^	0.652^a^

a *p < 0.05*.

## Discussion

In our study, the total PTG score among frontline nurses was 96.26 (SD = 21.57), which was at a high level. The results of this study are higher than those of other domestic scholars on post-traumatic growth of frontline nurses during the COVID-19 pandemic. The total PTG score of Cui's study (19) was 70.53 (SD = 17.26), Zhang's study (20) was 67.17 (SD = 14.79), and Li's study (21) was 70.40 (SD = 22.17). In the subscale of PTG, except for the scale of “Appreciation of Life,” the mean values of the other four scales were all higher than those of other scholars. This may be due to differences in the basic information of the participants. Nurses are more likely to develop PTSD than the general population because they are exposed to the frontlines of a disaster, and they are exposed to a stressful working environment and overwork (22). Furthermore, taking care of patients during the COVID-19 outbreak is a new challenge. COVID-19 patients die every day, possibly causing psychological shock and PTSD in nurses (23). Li et al. found that COVID-19 first responders had significantly higher PTSD scores than in healthcare workers who struggled with SARS in Zhang and Yang's survey (24–26). Meta-analysis provides evidence that PTG positively correlated with PTSD symptoms (27, 28). From this, we can preliminarily speculate that COVID-19 has had a significant post-traumatic growth for nurses working on the frontline.

Multiple regression analysis revealed that professional self-identity, social support, and whether nurses moved from other provinces to support Hubei Province were significant predictors of PTG. Notably, 214 (80.45%) nurses moved from all over China to support Hubei Province. In addition to China, the United States (29) and Peru (30) also called on nurses to join the fight against the epidemic and support the worst-affected areas. Medical workers faced cross-cultural adaptation problems, which were manifested in psychological and physiological adjustments in diet, living environment, and new working environment (31). Nurses not only take care of patients at risk of infection but also need to overcome cultural differences. Social and cultural maladjustment was a challenge for aid nurses (32). Previous studies have shown that highly challenging life events, circumstances, major life crises, trauma, and other extremely stressful events provoke an ability to cope with adversity, heighten self-discipline, and raise appreciation for life, bringing forth significant positive changes (33–36). Thus, cultural differences may be the reason for higher PTG scores in aid nurses. Research suggests that standardized multicultural nursing knowledge and skills training can improve the multicultural nursing ability of nurses (37). Good communication and psychological development between old and new team members can also help aid nurses understand the local situation and prepare for it, improving their ability of cross-cultural adaptation.

This study indicated that the professional self-identity of aid nurses was at the medium level (MD = 115.30, SD = 20.82 points). A significant positive correlation was found between nurses' professional identity and PTG (the *r* values were 0.720), that is, nurses with a high level of professional identity obtain high PTG, were highly likely to realize the value of their work, and gain a considerable sense of accomplishment from participating in COVID-19 epidemic treatment. Gibbons et al. found that job satisfaction and feeling valued in one's professional role were significant predictors of PTG in healthcare providers (38). Thus, our conclusions are consistent. All the nurses in this study volunteered to fight against the epidemic and did not regret this decision. Aylward noted that Chinese medical workers in the fight against the COVID-19 epidemic have a sense of responsibility and collective action; they all had a mindset of fighting to complete the task (39).

It was found that there was a significant positive correlation between nurses' social support and PTG (the *r* values were 0.620), indicating that nurses with high level of social support could obtain high PTG. Our findings indicated that social support should be a predictor of post-traumatic growth, consistent with other studies. Social support and empathy (40), cumulative exposure to traumatized patients (41), therapists' bonds with their patients (42), and professional self-esteem and secondary traumatization (43) have been identified as significant predictors of PTG in healthcare providers. Attachment theorists had suggested that viewing others favorably had profound influences on social relations (44). Social support is an important protective factor for psychological resilience that maintains mental health and lifts psychological barriers (45). Given the high infectivity of COVID-19, to reduce cross-infection, nurses need to stay alone in a single room when they finish working. They may feel loneliness and helplessness. Thus, efforts to improve social support systems of nurses who work against COVID-19 are necessary.

Several limitations should be considered. Firstly, this survey only investigated the nurses who were fighting against COVID-19 in Hubei Province without data from nurses from other provinces. Secondly, as a cross-sectional design, this study could only evaluate PTG at a specific time without longitudinal observation of the subjects. Thirdly, due to time constraints, we only conducted a questionnaire survey and not an intervention. Fourthly, this study did not assess personality traits involved in PTG and professional identity.

## Conclusions

PTG in the nurses who faced COVID-19 was found to be at an above-average level. Nurses from other provinces moving to support Hubei Province, professional self-identity, and social support were important influencing factors. Nurse leaders should pay attention to PTG and the influencing factors of nurses and offer solutions to retain mental health among these nurses.

## Data Availability Statement

The dataset compiled for this study is available upon reasonable request to the corresponding authors.

## Ethics Statement

The studies involving human participants were reviewed and approved by the Ethics Committee of The Second Affiliated Hospital of Guangxi Medical University (No. 2020-KY0005). The patients/participants provided their written informed consent to participate in this study.

## Author Contributions

HH and RL conceived and designed this study. YM and PT created and performed the literature search strategy. LC and GLiu built the data extraction file. SL and GZ conducted the data extraction. GLi and YM gathered the results. All authors contributed extensively to this work, interpreted the data and contributed substantially to the writing and revision of the manuscript, and read and approved the final manuscript.

## Funding

This work was supported by funds from the Key Research and Development Project of Guangxi (Grant number: 2020AB39028), Innovation Project of Guangxi Graduate Education (Grant number: JGY2020053), Medical Specialty Working Committee of the Chinese Association for Academic Degrees and Graduate Education (Grant number: B1-YX20190304-0), Scientific Research Project of Guangxi Health Commission (Z20201402), and Scientific Research Project of the Second Affiliated Hospital of Guangxi Medical University (EFYKY2020013).

## Conflict of Interest

The authors declare that the research was conducted in the absence of any commercial or financial relationships that could be construed as a potential conflict of interest.

## Publisher's Note

All claims expressed in this article are solely those of the authors and do not necessarily represent those of their affiliated organizations, or those of the publisher, the editors and the reviewers. Any product that may be evaluated in this article, or claim that may be made by its manufacturer, is not guaranteed or endorsed by the publisher.
